# Poly(ADP-Ribose) Polymerase Inhibitors in Pancreatic Cancer: A New Treatment Paradigms and Future Implications

**DOI:** 10.3390/cancers11121980

**Published:** 2019-12-09

**Authors:** Medhavi Gupta, Renuka Iyer, Christos Fountzilas

**Affiliations:** 1 Department of Medicine, Roswell Park Comprehensive Cancer Center, Buffalo, NY 14263, USA; Medhavi.Gupta@RoswellPark.org; 2 Department of Medicine/Division of GI Medicine, Roswell Park Comprehensive Cancer Center, Buffalo, NY 14263, USA; Renuka.Iyer@RoswellPark.org

**Keywords:** biomarkers, pancreatic neoplasms, poly(ADP-ribose) polymerase inhibitors

## Abstract

Pancreatic ductal adenocarcinoma (PDAC) is an aggressive malignancy. Most of the patients of PDAC present at later stages of disease and have a five-year survival rate of less than 10%. About 5–10% PDAC cases are hereditary in nature and have DNA damage repair (DDR) mutations such as *BRCA 1* and *2*. Besides having implications on screening and prevention strategies, these mutations can confer sensitivity to platinum-based therapies and determine eligibility for poly(ADP-ribose) polymerase inhibitors (PARPi). In the presence of DDR mutations and PARPi, the cells are unable to utilize the error-free process of homologous recombination repair, leading to accumulation of double stranded DNA breaks and cell death eventually. Various PARPi are in clinical development in PDAC in different subgroup of patients as monotherapies and in combination with other therapeutics. This review would focus on the mechanism of action of PARPi, history of development in PDAC, resistance mechanisms and future directions.

## 1. Introduction

Pancreatic ductal adenocarcinoma (PDAC) cancer is an aggressive malignancy. In United States, it is currently the third leading cause of cancer-related mortality [1]. Most patients have advanced (locally advanced unresectable or metastatic) disease on presentation [2] and despite advances in development of multiagent cytotoxic regimens [3,4,5,6,7], the overall five-year overall survival (OS) is less than 10% [1]. There is an unmet need to develop new treatment strategies. Personalized, biomarker-based options for patients with advanced PDAC do exist, i.e., pembrolizumab for microsatellite instability-high (MSI-H)/mismatch repair deficient (d-MMR) [8] and larotrectinib for tumors with neurotrophic receptor tyrosine kinase (NTKR) gene fusions [9], but this accounts for <1% of total patient population.

Approximately 5–10% PDAC cases are hereditary in nature and are associated with germline mutations in *BRCA 1* and *2*, *ATM, CDKN2A, STK11* (Peutz-Jeghers syndrome) and *MLH1, MSH2, MSH6, PMS2*, and *EPCAM* (Lynch syndrome) [10,11,12,13]. Approximately 4–7% of patients with PDAC have germline *BRCA1*/*2* mutations (*gBRCA1/2*) [14,15]. Beyond screening and potentially early detection, identification of these mutations has potential therapeutic implications as they confer increased sensitivity to platinum-based chemotherapy and poly(ADP-ribose) polymerase inhibitors (PARPi) [16]. Specifically, PARPi lead to unrepaired accumulation of single strand DNA breaks (SSBs) that eventually culminate into double strand breaks (DSBs), which, in the presence of *BRCA1/2* mutations and resulting deficiency in the homologous recombination (HR) repair mechanism, remain unrepaired, leading to cell death [17].

In this review, we would discuss the mechanism of action of PARPi, clinical applications in advanced PDAC, resistance mechanisms as well as opportunities for future development of these agents in PDAC.

## 2. Mechanism of Action of PARP Inhibitors

### 2.1. Repair Mechanisms in Healthy Cells

DNA damage through exogenous and endogenous stressors is common in normal cells. DNA damage repair (DDR) occurs via four major pathways; nucleotide excision repair (NER), base-excision repair (BER), MMR, HR, and non-homologous end joining (NHEJ) [18]. SSBs are repaired using the complimentary healthy strand mainly by BER, whereas DSBs repair occurs through HR and NHEJ [19,20]. BER pathway is majorly mediated by a class of 17 enzymes known as PARP [18]. These enzymes utilize NAD+ as a substrate to polymerize ADP-ribose units (PARylation), releasing nicotinamide as a by-product [21,22]. PARP1 is the important part of the superfamily involved in BER [23].

**Figure 1:**
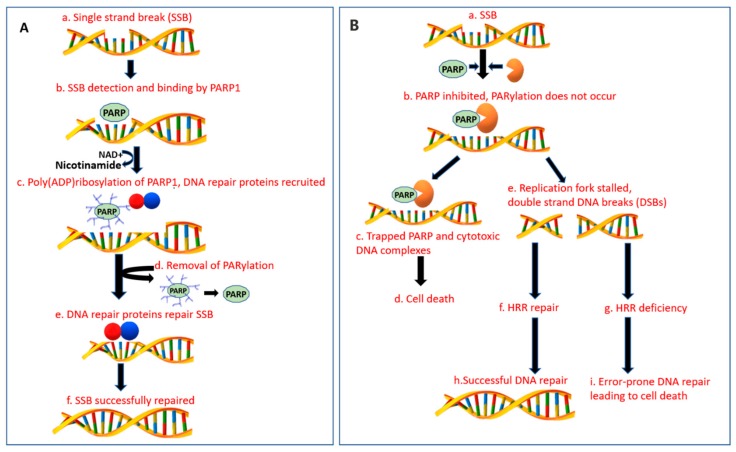
Mechanism of action of DNA damage repair in normal healthy cells and with PARP inhibitors. Abbreviations: SSB = single strand break, DSB = double strand break, DDR = DNA damage repair, HRR = homologous recombination repair, PARP = poly(ADP-ribose) polymerase, PARPi= PARP inhibitor, BER = base excision repair. (**A**) Normal DNA damage repair process: (**a**) In healthy cells, SSB mainly by BER pathway mediated by a family of enzymes known as PARP. (**b**) PARP-1 detects SSBs and binds to the DNA damage site via zinc motif fingers at the DNA binding domain. (**c**) PARP DNA binding activates its catalytic activity and utilization of NAD+ to synthesize poly ADP-ribose (pADPr) polymer formation on itself (autoPARylation) and other histone proteins. The pADPr polymers recruit DNA repair proteins, including XRCC1. (**d**) PARylation also reduces the affinity of PARP-1 for DNA binding, releasing it from the site of DDR. The pADPr polymers are lysed from PARP by enzymes such as PARG and ARH3, restoring its ability to detect and bind to DNA damage sites. (**e**) PARP removal from the site of DNA damage allows DDR effector proteins to bind at the site of damage leading to successful repair of DNA as depicted in (**f**). (**B**): (**a**) SSB in the presence of PARPi. (**b**) PARPi bind with the catalytic domain of PARP enzyme and inhibit the synthesis of pADPr formation and recruitment of DNA repair proteins. (**c**) PARPi also trap PARP-1 on the damaged DNA site, prevent its release and accumulate cytotoxic DNA complexes. (**d**) This eventually can culminate in cell death. (**e**) When BER mechanism is dysfunctional, unrepaired SSBs stall the replication fork leading to DSBs formation. (**f**–**i**) The concept of synthetic lethality. (**f,h**) In *BRCA 1 or 2* heterozygous (*BRCA*^+/−^) or *BRCA wild-type* (*BRCA*^+/+^) cells, the DDR occurs through the error-free HRR pathway averting the cell death. (**g**) In the presence of BRCA1 or BRCA2 mutations (*BRCA*^−/−^) and other mutations with a similar phenotype of defective HRR, the cells are unable to repair DSBs through HR pathway. (**i**) Repair alternatively occurs through the error-prone pathway of NHEJ leading to cell cycle arrest, genomic instability and lethality.

PARP1 has three functional domains: a DNA binding domain that facilitate binding to SSBs and DSBs, an automodification domain that accepts ADP-ribose units for poly ADP-ribose formation and a catalytic domain involved in transferring ADP-ribose from NAD+ to protein acceptors [20]. PARP1 detects the SSBs and binds to the site of damage via zinc finger motif (Figure 1A). This leads to activation of its catalytic activity, leading synthesis of poly ADP-ribose, that subsequently recruits various DRR proteins such as XRCC1 [24,25] and reduces the affinity of PARP-1 for DNA, facilitating its release, to allow binding of other DDR proteins [26,27]. Enzymes such as PARG and ARH3 lyse poly(ADP-ribose) from PARP1 for restoration of its function [28,29].

When SSB repair mechanisms are dysfunctional, unrepaired SSBs accumulate at the replication fork (RF) leading to DSBs formation that is repaired by HR and NHEJ (Figure 1B). HR is a conservative mechanism that repairs the DNA breaks precisely upon availability of sister chromatid DNA, thereby maintaining the genetic stability [19]. BRCA1/2 work in conjunction to facilitate DDR via HR. BRCA2 facilitates translocation of DNA repair protein Rad51 to the site of DNA damage [30,31]. BRCA1 works upstream by signaling response to damage, acts as a cell cycle checkpoint regulator and recruits repair enzymes [32]. Besides its role in SSB repair, PARP1 is also involved in orchestration of HR via recruitment of mitotic recombination 11 (MRE11) and ataxia telangiectasia-mutated (ATM) components needed to restart stalled RFs. When the undamaged sister chromatic DNA is unavailable, DSB repair occurs through a quicker and a less precise mechanism of NHEJ which increases the chances of DNA rearrangements and genetic instability [20,33]. PARP1 has also implications in NHEJ and transcription modulation [34,35].

In the presence of PARP inhibition or deficiency, cells are unable to repair SSBs. Aggregation of unrepaired SSBs stalls the RF, leading to either RF collapse or conversion of SSBs to DSBs [36]. The HR pathway is activated as a compensatory mechanism, leading to a quick, precise and an effective DDR.

### 2.2. BRCA Deficiency

*BRCA1* (located on 17q21) and *2* (located on 13q12.3) are integral for genome stability by facilitating HR as mentioned above, and have an autosomal dominant pattern of inheritance with an incomplete penetration [30]. There are more than 1600 mutations associated with *BRCA1/2*, however, not all of them are considered pathogenic. The majority of the deleterious mutations leading to BRCA deficiency and subsequently HR deficiency (HDR) are insertions, frameshift mutations or nonsense mutations. Different ethnicities have a different prevalence of these mutations (i.e., most Ashkenazi Jews have one of the three founder mutations in either *BRCA1* 185delAG or 5382insC or *BRCA2* 6174delT) [37]. Cells with *BRCA1/2* loss-of-function mutations cannot repair DSBs via HR but utilize NHEJ which could lead to accumulation of genetic alterations and ultimately lead to genetic instability or cell death [25]. Consequently, the presence of these mutations has been associated with increased risk of malignancies, including breast, ovarian and PDAC amongst others [38,39].

### 2.3. DNA Repair with BRCA-Deficient Cells in the Presence of PARPi

Utilizing PARPi in BRCA mutant malignancies is one of the first clinical applications of the age-old concept of synthetic lethality [40]. Synthetic lethality was described as a phenomenon in which a combination of two defects leads to cell death, but singularly neither of them has a detrimental effect individually [41]. As described earlier, in the presence of PARPi the cells are unable to repair SSBs with transformation to DSBs. In BRCA1/2-deficient cells, because of HRD, DSBs are repaired with either NHEJ or alternative-NHEJ, consequently leading to cycle arrest, DNA instability, and lethality (Figure 1B) [41]. Normal cells on the other hand, utilize the functional BRCA protein and are able to repair DSBs, leading to cell survival. Therefore, PARPi are able to spare healthy cells, making them an ideal therapeutic agent in this setting. Besides the catalytic inhibitory properties, PARPi also trap PARP1 on damaged DNA and prevent its release and subsequent recruitment of DNA repair proteins, leading to formation of cytotoxic DNA complexes [42]. HRD is not limited in *gBRCA1/2*. A similar phenotype (*BRCAness*) can result from either somatic *BRCA1*/*2* mutations or defects in other DDR genes such as *PALB2*, *ATM*, *ATR*, and *FANC* that are involved in HR [43,44,45], as well as from epigenetic *BRCA* silencing via promoter hypermethylation [46]. Identification of mutations beyond *BRCA1/2* might be of importance as these tumors could also exhibit sensitivity to platinum-based regimens and PARPi [16,47,48]; however, the HRD score was neither predictive of a response nor survival in advanced PDAC patients treated with platinum-based therapy [49]. The significance of HRD in PDAC in terms of response to PARPi is an area of active investigation.

### 2.4. PARPi Pharmacology

PARP-1 is the most abundant enzyme of the PARP family and is involved in posttranslational modification of proteins involved in DNA repair. PARPi contain a carboxamide group that forms hydrogen bonds with serine hydroxyl and glycine backbone of the nicotinamide (NAD+) pocket site of PARP enzymes, mimicking NAD [50]. The adjacent benzene ring interacts with the tyrosine residue and makes π–π stacking interactions. Both PARP-1 and PARP-2 have identical NAD+ pockets and most PARPi inhibit PARP-1 and 2 enzymes similarly, with minor differences in selectivity [51]. In the presence of HRD due to *BRCA1/2* mutations, the cells are unable to repair the double strand DNA (dsDNA) breaks through HRR and resort to non-conservative methods of DNA repair such as NHEJ, leading to DNA alterations and genomic instability [20]. In addition, PARPi trap PARP-1 at the site of DNA damage, preventing auto-PARylation and PARP-1 release.

Currently, six different PARPi (olaparib, rucaparib, veliparib, niraparib, talazoparib, and pamiparib) have been in clinical development at different stages. The comparisons in pharmacology are summarized in Table 1 and elaborated in this section. Single agent cytotoxicity is not directly proportional to PARylation inhibition and could be explained by differences in PARP trapping. In general, the IC_50_ of PARPi falls in the low nanomolar range. Talazoparib is comparable to olaparib in catalytic activity in vitro (IC_50_: 4 vs. 6 nmol/L, respectively) and five-fold more potent compared to rucaparib (IC_50_: 21 nmol/L), however was ~100 times more potent at trapping PARP and in terms of toxicity [42]. Talazoparib has superior trapping activity followed by niraparib; veliparib has the least trapping activity [42,52]. The differences in the trapping activity can be explained by differences in molecular structure with talazoparib being the largest and least flexible molecule [53,54]. The clinical and preclinical data with pamiparib is limited as of now. In a xenograft breast cancer model, it was found to be over 10 times more potent than olaparib [55]. 

The differences in potency also correlate with their toxicity profiles [42,68]. The most common adverse events (AEs) are gastrointestinal, hematological and constitutional (fatigue). Even though, it is difficult to compare toxicities across different trials with heterogenous patient populations, there are few points worth noting. Rucaparib leads to inhibition of renal transporter proteins involved in secretion of creatinine and can lead to increased creatinine (any grade 15%, grade 3 ≤ 1%) [63]. Transaminitis is generally self-limiting, highest with rucaparib (any grade 34%, grade 3 = 10%) [63]. Hematological toxicities are the highest with niraparib (any grade-thrombocytopenia 61%, anemia 50%, neutropenia 30%, grade >/3- thrombocytopenia 34%, anemia 25%, neutropenia 20%) [64]. Toxicities are more common in the first few cycles of treatment, warranting closer early monitoring.

### 2.5. Clinical Development of PARPi in PDAC

The clinical evolution of PARPi in PDAC has evolved from being used as monotherapies in refractory disease, to maintenance therapies and in combination with other classes of therapeutics (Table 2). Contrasting the uniformly encouraging efficacy results during early development in ovarian and breast cancer between different PARPi, the reality has been different in PDAC with most patients not deriving or having a very short-term clinical benefit despite enrollment of a molecularly selected population, as we will discuss in this section of this review. Furthermore, the dual mechanism of action and the pharmacologic differences in between different agents makes evaluation of PARPi as a class challenging and does not allow easy incorporation into approved combination strategies in use in PDAC. Specifically, synergy with camptothecins appears to be mainly dependent on the inhibition of PARylation activity while for alkylating agents, the trapping activity is at least as important as PARylation inhibition [52]. In addition, due to overlapping toxicities with commonly used cytotoxic agents, the ability to use PARPi in combinations at doses that can achieve DNA trapping is limited [69]. 

### 2.6. PARPi as Monotherapy in Advanced Disease

One of the first clinical trials to evaluate the role of PARPi in metastatic PDAC patients was a phase II clinical trial involving olaparib monotherapy in patients with advanced recurrent cancers with *gBRCA1/2* mutations [56]. The PDAC cohort (23 patients) had a mean two prior lines of therapy with 65% having prior platinum-based therapy. Compelling single agent activity was noted with an overall response rate (ORR) of 22% regardless of platinum exposure. Responses were durable with stable disease (SD) >/8 weeks in 35% of patients and its was well tolerated.

The activity of rucaparib as single agent was tested in the RUCAPANC phase II trial involving both somatic *BRCA1/2* (*n* = 3) or g*BRCA1/2* mutant (*n* = 16) advanced PDAC patients [70]. Seventy-nine percent of patients were exposed to platinum and 42% were refractory to platinum. The study did not meet its primary endpoint with ORR of 16% and disease control rate (DCR) of 32%. None of the patients with response had progressed on prior platinum-based therapy and 3 out of the 4 patients with response had only one prior line of therapy.

Veliparib was evaluated in a phase II single arm trial in advanced, pre-treated PDAC patients, with a known g*BRCA1/2* or *PALB2* mutation, including platinum-resistant disease [71]. No radiological response was observed; SD was seen in 25% patients at 8 weeks.

First-in-human phase Ib trial (NCT01286987) of talazoparib was conducted in *gBRCA1/2* mutant patients in advanced refractory solid tumors including metastatic PDAC (n=13, median prior chemotherapy regimens and prior platinum regimens of 2.5 and 1, respectively). It was well tolerated and had an ORR of 20% and SD in 10% [76].

Two parallel phase II clinical trials in US and Israel (NCT02677038, NCT02511223) are testing olaparib in patients with >/1 lines of therapy in metastatic *BRCAness* PDAC, defined as either DDR deficiency beyond *gBRCA1/2* mutation or a family history of *BRCA*-associated cancers in the absence of DDR deficiency or ATM loss by immunohistochemistry. Preliminary results (45% enrollment) showed efficacy signal only in patients with platinum-sensitive disease [72].

### 2.7. PARPi as Maintenance Therapy in Platinum-Sensitive Disease

Based on available evidence, the window of opportunity for benefit from PARPi in BRCA-deficient PDAC appears to be as maintenance therapy in platinum-sensitive disease. The randomized, double-blind placebo-controlled phase III POLO trial evaluated the role of maintenance olaparib in patients with g*BRCA1/2* mutant metastatic PDAC, with non-progressive disease during first-line treatment with platinum-based chemotherapy for a minimum of 16 weeks [66]. Longer PFS with olaparib was observed (7.4 months) as compared to 3.8 months with placebo (HR 0.53; 95% CI 0.35 to 0.82; *p* = 0.004) and the study met its primary endpoint. The overall survival (OS) was similar (46% data maturity). The rate of grade 3 or higher treatment-related AEs with higher with olaparib (40%) compared to placebo (23%) with no deterioration of quality of life.

A single arm phase II study (NCT03140670) is evaluating the role of maintenance rucaparib advanced PDAC patients with either germline or somatic *BRCA1/2* or *PALB2* mutations, with non-progressive disease after at least 16 weeks of platinum-based regimen [73]. Early results are encouraging with a median PFS of 9 months and ORR of 37%. Clinical benefit was durable with DCR of 90% patients for at least 8 weeks.

NIRA-PANC is a phase II clinical trial (NCT03553004) assessing the role of maintenance niraparib after >1 line of therapy in metastatic PDAC patients with either germline or somatic HRD. No results have been reported so far [77].

## 3. PARPi in Combination with Other Therapies

### 3.1. PARP in Combination with Chemotherapy 

**Figure 2:**
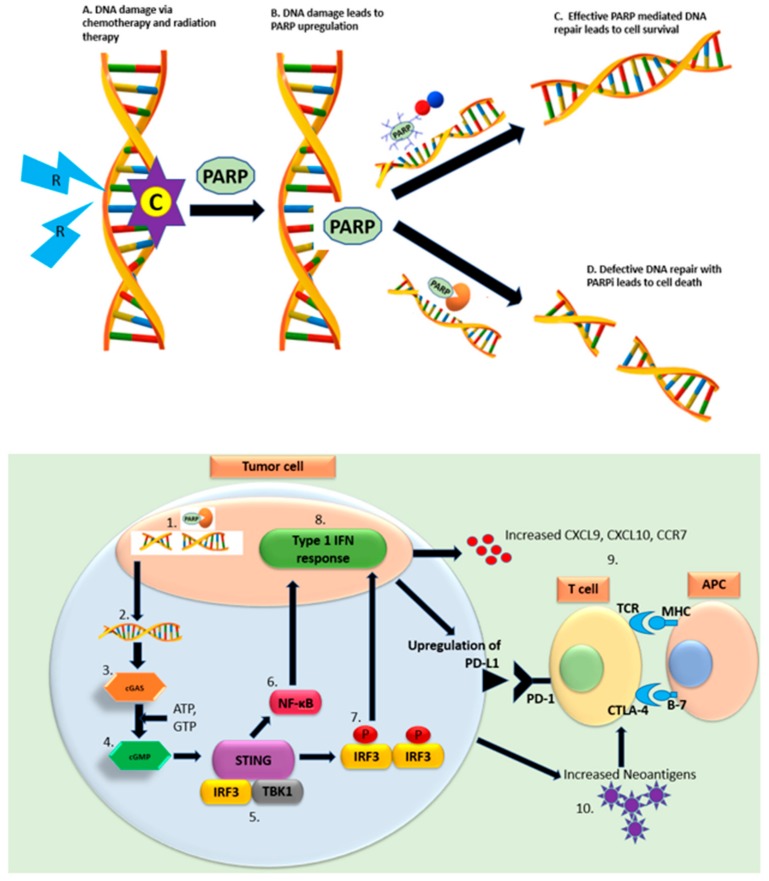
PARP inhibitors in combination with other therapeutics. Abbreviations: C = chemotherapy; R = radiotherapy; cGAS = cyclic GMP-AMP synthase; cGMP=cyclic guanosine monophosphate; STING = stimulator of interferon genes; IRF3 = interferon regulatory factor 3; TBK1 = TANK binding kinase 1; NF-κB = nuclear factor kappa-light-chain-enhancer of activated B cells; IFN = interferon. (Upper panel): PARP inhibitors in combination with chemotherapy and radiation therapy (**A**) Chemotherapy agents like platinum containing compounds and topoisomerase inhibitors and radiation therapy lead DNA damage. (**B**) DNA breaks lead to upregulation of PARP enzymes. (**C**) In normal healthy cells, PARP-1 binds to DNA damage site leading to poly ADP-ribose formation, recruitment of DNA repair proteins. This process leads to successful repair of DNA and cell survival. (**D**) PARPi binds to catalytic site PARP enzyme and traps PARP-1 at the NA damage site. This leads to defective DNA repair and eventually, cell death. (Lower Panel): (**1**) PARPi lead to double strand (ds) DNA breaks, (**2**) dsDNA breaks lead to generation of cytosolic DNA fragments, (**3**) Cytoplasmic dsDNA activates cGAS, (**4**) Activation of cGAS catalyzes production of cGMP, (**5**) cGMP activates STING pathway, (**6**–**8**) STING pathway in return activates either NF- κB or TBK1- IRF3-Type I IFN pathway. (**9**) Activation of Type I IFN pathway alters tumor immune microenvironment through upregulation of PDL-1 and MHC and increased CXCL9, 10, CCR7 leading to enhanced cytotoxic T cell, helper T cells and dendritic cell function. (**10**) Homologous recombinant deficient tumors secrete a higher neoantigen load that is associated with an increased number of tumor-infiltrating lymphocytes.

The rationale behind combining PARPi with chemotherapeutic agents has been illustrated in Figure 2A. Oxaliplatin has been shown to have synergistic antitumor activity with PARPi in vivo. Interestingly the combination prevented oxaliplatin-induced neurotoxicity [78]. Several clinical trials are evaluating PARPi with platinum-based therapy. A phase I/II clinical trial (NCT01489865) investigated the combination of veliparib with mFOLFOX6 (folinic acid+ 5-FU+ oxaliplatin) in metastatic, pre-treated PDAC patients [75]. Patients in phase II (*n* = 33) were pre-selected for germline or somatic DDR mutations (69%) or have a family suggestive of a breast or ovarian cancer syndrome (BOCS, 27%) and included both pretreated (18 patients) and untreated (15 patients). The ORR for the whole cohort was 26%. ORR was higher in platinum-naïve (33%) vs. platinum pretreated (7%), patients with BOCS (30%) vs. not (14%), and DDR mutation positive (50%) vs. negative (17%). The ORR for the platinum-naïve, DDR mutation positive, BOCS cohort was 58% highlighting the importance of patient selection. The median PFS and OS for these patients was 8.7 and 11.8 months respectively (all patients, PFS 3.7 months/OS: 8.5 months). The combination was well tolerated with grade >/3 treatment-related AEs of myelosuppression (16%) and nausea, vomiting (6%). Veliparib with oxaliplatin/capecitabine in patients with a known *BRCA1*/*2* mutation or *BRCA*-related advanced malignancies was also found to be safe in a phase I clinical trial (NCT01233505) [79]. Whether the incorporation of PARPi in a platinum backbone really leads to improved outcomes (vs. using as maintenance) remains to be seen. A phase II trial (NCT01585805) is enrolling *BRCA/PALB2* mutated advanced PDAC patients in the front-line setting comparing gemcitabine/cisplatin with and without veliparib, and will help answer this question [80].

Topoisomerase inhibitors (TIs) stall the RF by stabilizing the DNA complex in unrepaired state, enhancing SSBs [81]. Studies showed that PARPi can enhance the cytotoxicity of TIs at concentrations lower than the ones necessary for PARP synthesis in HRD cells [82]. SWOG S1513 (NCT02890355) randomized mPDAC patients to veliparib plus modified FOLFIRI (Folinic acid + 5-FU + Irinotecan) vs. FOLFIRI alone as second-line treatment in a biomarker unselected population [74]. Nine percent of patients had mutation in HRD gene and 20% had mutations in other DDR genes. Interim analysis showed that the combination arm did not have an OS benefit (5.1 vs. 5.9 months; HR 1.3, 95% CI 0.9–2). Similarly, there was no benefit from the addition of veliparib in patients with HRD or non-HRD mutations (OS 7.4 vs. 9.4 months in FOLFIRI alone). The incidence of grade >/3 treatment-related AEs like neutropenia, fatigue, and nausea was higher in the veliparib arm [74]. A phase I/II trial (NCT03337087) is investigating rupacarib along with liposomal irinotecan, leucovorin, 5-FU in gastrointestinal malignancies including PDAC, with preselection in phase II portion in PDAC cohort for HRD (*BRCA* or *PALB2 mutated* or *HRD non BRCA/PALB2* non-mutated). Gemcitabine in combination with PARPi is more cytotoxic against PDAC in vitro compared to gemcitabine alone [83]. A phase I study (NCT00515866) is exploring the safety of olaparib with gemcitabine in the front-line setting with advanced solid tumors, including advanced PDAC, without any selection for specific mutations [84].

### 3.2. PARP in Combination with Radiotherapy

PARPi were found to have radiosensitizing effects in vitro [85]. Pretreatment with rucaparib followed by gemcitabine and radiation therapy increased cytotoxicity by stalling the cells in G2/M phase leading to apoptosis and cell death. Furthermore, in vitro and in vivo experiments showed that veliparib in combination with radiation led to a better tumor growth inhibition and survival, rather than single-agent activity of either of the two agents [86]. Veliparib inhibited polymerization of PAR protein with compensatory upregulation of p-ATM and PARP with enhanced DNA damage and apoptosis. The combination of PARPi and radiation therapy with or without chemotherapy is being investigated in two clinical trials. Niraparib is being tested in a phase II proof of concept trial (NCT03601923) in patients with advanced PDAC harboring HRD germline or somatic mutations (*BRCA1*, *BRCA2*, *PALB2*, *CHEK2*, or *ATM* mutations) in the second line setting, with exclusion of patients with progressive disease on oxaliplatin-based regimens. Palliative radiation therapy to be administered 1 week before the start of niraparib. Veliparib is being evaluated in a phase 1 clinical trial (NCT01908478) in patients with locally advanced unresectable or borderline resectable PDAC in combination with gemcitabine and intensity modulated radiation therapy in the front-line setting with no selection for mutations.

### 3.3. PARPi in Combination with Immunotherapy

The synergy between PARPi and immunotherapy is likely multifactorial as shown in Figure 2B. In a high-grade serous ovarian carcinoma model, HRD tumors harbored a higher neoantigen load with an increased tumor-infiltrating lymphocytes (TILs) and PD-1/PD-L1 expression as compared to non HRD tumors (Figure 2B, 10) [87]. Besides the enhanced neoantigen load, S-phase DNA damage induces activation of PD-L1 through stimulator of interferon genes (STING) pathway [88]. Exposure of double stranded DNA in cytoplasm activates cyclic GMP-AMP synthase (cGAS) and catalyzes production of cyclic-dinucleotide (CDN) (Figure 2B, 2–4), which activates STING with subsequent activation of either the NF-κB or the TBK1- IRF3-Type I IFN pathways (Figure 2B, 5–8) [89]. Type I IFN have immunostimulatory effects such as upregulation of MHC and CCR7 leading to enhanced dendritic cell function [90], increase in T-helper cells chemokines (CXCL9 and CXCL10) [88] and potentiation of cytotoxic T-cell lymphocyte function (Figure 2B, 9) [91]. Furthermore, it suppresses regulatory T-cells (T_reg_) cells by downregulating cyclic AMP (cAMP) [92]. PARPi upregulated PD-L1 expression in an in vivo breast cancer model via GSK3β inactivation. Simultaneous treatment with PD-L1 blockade and olaparib led to enhanced T-cell mediated killing of tumor cells [93]. Similarly, in a BRCA1-deficient ovarian cancer model, PARPi led to activation of STING pathway and the therapeutic efficacy was improved with PD-1 blockade [91]. Interestingly, synergistic anti-tumor activity of niraparib and PD-1 blockade was seen in both BRCA-deficient and BRCA-proficient immunocompetent tumor models indicating that this combination may be active regardless of BRCA status. Furthermore, mice in complete remission after treatment rejected implanted tumor cells indicating the generation of memory T-cells [94]. Preclinical studies have shown that DSBs leads to upregulation of PD-L1 expression through ATM/ATR/Chk-1. Furthermore, treatment with radiotherapy/PARPi in BRCA2-depleted cells leads to enhancement of Chk-1-dependent PD-1 upregulation [95]. The therapeutic efficacy of PARPi and Chk-1 inhibitors with PD-L1 blockade was seen in a small cell lung cancer carcinoma model through activation of STING pathway [96]. The data of combination of PARPi with anti-CTLA-4 is limited. Therapeutic efficacy of the combination was seen in a BRCA1-deficient ovarian cancer model [97].

A large number of clinical trials are exploring the combination of immunotherapy and PARPi across various tumor types. Preliminary data from a phase II study (NCT02484404) in unselected metastatic prostate cancer showed that the combination of olaparib and durvalumab is efficacious (8/17 or 47% patients had PSA responses >50%) and is tolerable [98]. A phase II (MEDIOLA) trial in relapsed gastric cancer with a 4-week run in of olaparib, followed by combination with durvalumab failed to show efficacy (DCR at 12 weeks was 26%) due to early PD during the run-in period [99]. Combination of niraparib and pembrolizumab in a phase II trial (TOPACIO/Keynote-162) in triple negative breast cancer (TNBC) and recurrent ovarian cancer patients showed that the combination is safe and effective, irrespective of the platinum exposure or BRCA 1/2 or PD-L1 status [100,101]. A phase I study involving a combination of olaparib, durvalumab, and the vascular endothelial factor receptor (VEGFR)-1 inhibitor cediranib in ovarian/endometrial/TNC patients showed that the combination is safe; there was an efficacy signal with DCR of 67% [102]. The ongoing phase Ib/II trial PARPVAX study (NCT03404960) evaluates niraparib with either nivolumab or ipilimumab as maintenance therapy in patients with advanced, platinum-sensitive PDAC [103].

### 3.4. Molecular Targeted Therapy Combinations

Preclinical data has suggested synergism between PARPi and MEK inhibitors in *RAS*-mutant cells. A study involving PDAC cells showed that the synergy is mediated through multiple mechanisms including increased expression of FOXO3a leading to apoptosis, decrease in HR ability, increased PARP protein, enhanced PARPi induced DNA damage, and increased hypoxia through decreased vascularity [104]. The combination was effective in cells without *BRCA* mutations. One of the phase II feasibility studies (NCT04005690) would entail administering olaparib or MEK inhibitor cobimetinib in patients with resectable PDAC in the neoadjuvant setting, with biomarker evaluation before and after therapy. A phase Ib/II study is evaluating talazoparib in combination with avelumab and binimetinib in patients with *NRAS/KRAS* mutant PDAC and other advanced solid tumors after previous 1–2 lines of therapy.

Preclinical data has shown anti-angiogenic effect of PARPi through inhibition of endothelial cell migration at concentrations lacking cytotoxic effects, suggesting a novel therapeutic implication of PARPi [105]. A single arm phase II clinical trial (NCT02498613) is exploring the combination of olaparib with the VEGF inhibitor cediranib in patients with advanced solid tumors including advanced PDAC in genetically unselected population. The SMMART trial (NCT03878524) is an open-label biomarker-driven phase Ib trial in patients with PDAC, breast, prostate cancer, and acute myeloid leukemia involving administration of a combination of two drugs from 35 experimental therapeutics, including olaparib based on molecular testing.

## 4. Resistance Mechanisms

**Figure 3:**
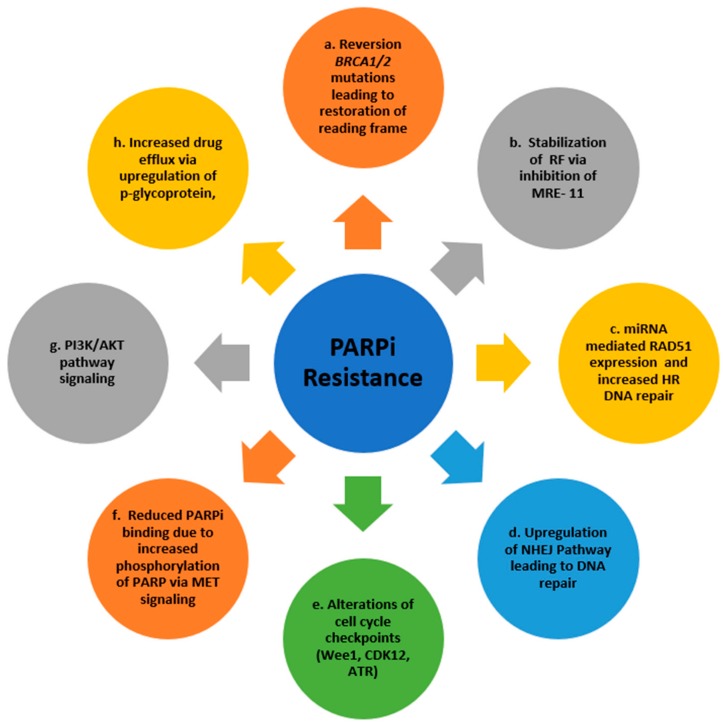
Mechanisms of resistance to PARPi. Abbreviations: RF = replication fork; MRE 11 = mitotic recombination 11; miRNA = microRNAs; HR = homologous recombination; NHEJ = non-homologous end joining; PI3K = phosphatidylinositol 3-kinases. Cells with HRD can acquire resistance through upregulation of an alternative-NHEJ error-prone pathway, known as microhomology-mediated end-joining (MMEJ) repair. MMEJ repair requires DNA polymerase POLQ for repair of DSBs [109]. Preclinical data suggests that therapeutic targeting by POLQ inhibition can have synthetic lethality effect in HRD cells [109,110].

The various mechanisms through which tumor cells acquire resistance to PARPi have been illustrated in Figure 3. Several studies suggested that acquired *BRCA* mutations in patients with *gBRCA1/2* could lead to restoration of HR and confer resistance of platinum agents and PARPi. An in vitro study by Sakai et al. [106] demonstrated that intragenic mutations in *BRCA2* mutant PDAC and ovarian carcinoma cell lines restore the *BRCA2* reading frame leading to both cisplatin and PARPi. Norquist et al. [107] showed that these secondary reversion mutations were found in 29% of *gBRCA1/2* mutant recurrent ovarian carcinomas and were predictive of resistance to platinum containing chemotherapy and PARPi. A UK study [108] demonstrated olaparib resistance with emergence of a secondary *BRCA2* mutation with restoration of its function.

In the absence of *BRCA2* reversion mutations, tumor cells can evade lethality by PARPi through protection of RF. Chaudhuri et al. [36] showed that deficiencies of MLL3/4 in *BRCA2* mutant cells inhibits the recruitment of MRE11 to the RF in vitro, thereby preventing the RF degradation. Furthermore, 53BP1 regulates the balance between HRR and NHEJ [111]. 53BP1 loss downregulates NHEJ and promotes error free HR, thereby conferring resistance of PARPi [111]. Finally, microRNAs can modulate sensitivity to PARPi. For example, miR-107 and miR-222 downregulate expression of RAD51, thereby impairing DDR by HR and enhancing sensitivity to olaparib [112]. In addition, miR-622 mediated resistance to PARPi and cisplatin in *BRCA1* mutant cells by diverting the repair towards HR pathway and suppressing the NHEJ via Ku complexes [113].

DDR is dependent on the phase of cell cycle; modulating cell cycle checkpoints can potentially alter the effect of PARPi in tumor cells. Inhibition of CDK12 leads to downregulation of DDR genes [114] and reverses resistance to PARPi in breast cancer [115] and multiple myeloma in vitro [116]. PARPi-induced DNA damage can activate the ATR-mediated G2/M checkpoint facilitating DNA repair which can be reversed by ATR inhibition leading to cell death [117]. Similarly, Wee1 inhibition can allow unrepaired DNA enter mitosis via the G2/M checkpoint but the results of phase I study with olaparib with Wee1 inhibitor in unselected were disappointing with ORR of only 11% [118]. MET phosphorylates PARP1 at Tyr907, increases its enzymatic activity thereby reducing the affinity for PARPi and leads to resistance [119]. PARPi in combination with a MET inhibitor can overcome PARPi resistance in vitro [120]. The phosphoinositide 3-kinase (PI3K) signaling pathway has a role in tumorigenesis through maintenance of HR steady state [121]. A study in TNBC showed that PI3K inhibition leads to sensitization to PARPi by downregulation of *BRCA1/2*, increased poly ADP-ribosylation and DNA damage [122]. Finally, in BRCA-deficient cells, resistance due to upregulation of genes responsible for p-glycoprotein with subsequent increased efflux of PARPi can develop, which can be counteracted by p-glycoprotein inhibitors [123]. A next generation PARPi (AZD2461), that is not a substrate for p-glycoprotein is in development and is thought to potentially overcome p-glycoprotein-mediated olaparib resistance [124].

## 5. Discussion

Advanced PDAC is one of the most challenging malignancies to treat. PDAC is catching up with the current wave of precision medicine with the goal of improving patient outcomes. Besides two tissue-agnostic therapies (pembrolizumab for MSI-H/d-MMR and larotrectinib for NTRK fusion gene positive tumors), PARPi are an exciting addition to our armamentarium of drugs for patients with pathogenic *gBRCA1/2* mutations and olaparib is endorsed by the National Comprehensive Cancer Network (NCCN) as appropriate maintenance therapy after at least 4 months of platinum-based therapy provided that there is no interim disease progression [125]. With the advent of emerging clinical data, there are several questions that remain answered.

Firstly, whether the benefit seen in the *gBRCA* mutant patients in POLO trial could be extrapolated to tumors with somatic *BRCA* mutation or in patients with other HRD or DDR mutations remains unclear. The one patient with somatic *BRCA2* and the two patients with *gPALB2* mutations enrolled in the maintenance rucaparib study so far (24 out of 42 planned patient accrual) attained an objective response indicating that the benefit of PARPi potentially extends beyond *gBRCA1/2* [73]. Data from a large number of clinical trials (NCT02042378, NCT02677038, NCT02511223, NCT03140670, NCT02890355, NCT01489865) evaluating the role of PARPi in patients with somatic mutations would provide further clarity regarding the extent and durability of response in this group of patients. The NCCN endorses testing patients for infrequent but potentially actionable somatic alterations including *BRCA1/2* mutations [125]. Whether archival or fresh tissue should be used for somatic mutation screening is unclear. Data from retrospective ovarian cancer tumor sample analysis suggests that BRCA1/2 loss occurs early in the course of the disease [126] but it is not currently known if this is true in PDAC. Validation of predictive biomarkers beyond *gBRCA1/2* mutations, NTKR fusions, and MMR/MSI status is an unmet need in PDAC.

Secondly, the appropriate timing of the use of PARPi in the disease course of PDAC is unclear. As mentioned in the sections earlier, PARPi are being tested both as monotherapy (maintenance and refractory) and in combination with other agents (front-line and subsequent lines of treatment).

In the maintenance setting, olaparib led to PFS benefit in g*BRCA1/2* platinum sensitive patients in POLO trial [66]. However, it is important to note that the preliminary data suggest no OS benefit and it is possible that use of PARPi in subsequent lines of therapy can be as beneficial as in the maintenance setting, but whether this is dependent on platinum sensitivity is unclear. Rucaparib in patients with somatic or germline *BRCA1/2* mutations [70] and olaparib in patients with *BRCAness* [72] showed benefit only in platinum-sensitive disease, while with olaparib in patients with *gBRCA1/2*, the benefit was seen irrespective of the platinum-exposure [56]. Unfortunately, the initial report of the POLO study does not mention subsequent use of PARPi in patients treated with placebo. In addition, whether PARPi is superior to 5-FU maintenance as used in the PANOPTIMOX study is unknown [127]. There is currently no head-to-head comparison between maintenance PARPi and chemotherapy. Also, the appropriate time to switch therapy to PARPi is unclear. In the POLO trial [66], one-third of patients received platinum-based therapy for more than 6 months. On subgroup analysis for PFS, patients who received platinum-based therapy for >6 months seemed to have a greater benefit (HR 0.35, 95% CI 0.17–0.72) as compared to 4–6 months (HR 0.69, 95% CI 0.43–1.12). Finally, it is unclear if there is any benefit to platinum re-induction after progression on PARPi. Preclinical data reveal cross-resistance between PARPi and platinum chemotherapy [128] and, in an unselected patient population in the PANOPTIMOX study, reintroduction of FOLFIRINOX (folinic acid + 5-FU + irinotecan + oxaliplatin) upon disease progression led to a marginal benefit of 1.4 months in terms of median PFS [PFS1 (defined as time to first progression of disease) of 5.7 mo, PFS2 (defined as time to disease progression during FOLFIRINOX) of 7.1 mo [127]. Using an alternative alkylating agent such as mitomycin C, might be preferable to platinum re-induction but only anecdotal evidence exist so far [129]. Furthermore, there is no data to support switching from one PARPi to another at the time of disease progression.

Is it time to start using PARPi in combination with chemotherapeutic agents, be it in the first or subsequent lines of treatment? Though the mFOLFOX-6 plus veliparib combination was promising, especially in highly selected patients who are platinum-naïve and have DDR mutation and BOCS history, the lack of direct comparison to chemotherapy limits the upfront use of this strategy [75]. As a cautionary tale, SWOG S1513 showed detrimental effects for the combination, even for the subgroup of patients with DDR mutations [74]. Both trials showed an increased toxicity profile with the combination arms, indicating that the benefit of the combination (if at all) would potentially come at the cost of an increased toxicity [74,75]. The direct comparison of gemcitabine/cisplatin with and without veliparib in front-line setting in *BRCA1/2/PALB2* mutated PDAC will provide further insight [80]. Several other clinical trials are evaluating PARPi in combination with different chemotherapies in front-line (NCT01282333) or subsequent lines of therapy (NCT03337087, NCT01233505, NCT00515866).

Though relatively well tolerated, PARPi do have a toxicity profile that needs to be kept in mind while making therapy decisions and it is important to understand that different PARPi differ in their potencies for catalytic inhibition, PARP binding, and inhibition of PARylation; and hence, have differing toxicity profiles and potentially efficacy and therefore, cannot be assumed that can be used interchangeably. For example, rucaparib leads to elevated creatinine based on inhibition of renal transport proteins and has the highest rate of transaminitis [63]. On the other hand, niraparib has the highest rate of bone marrow suppression and resultant hematologic toxicities [64]. In POLO study [66], the PFS benefit came at the cost of increased incidence of grade 3 AEs. Even though there was no notable deterioration in QoL, it is worth noting that majority (67%) of the patients had excellent performance status (ECOG 0). In the real world, the PFS benefit could come at the cost of increased toxicity profile and QoL deterioration, as majority of PDAC patients do not have preserved performance status.

Beyond early identification of patients with *gBRCA1/2* pathogenic mutations, how can we better select patients for PARPi maintenance after platinum therapy? The development of reversion somatic *BRCA2* mutations during platinum-based therapy that limits the efficacy of PARPi. Tumor mutational profiling using next generation sequencing after completion of platinum induction can assist in identifying patients with secondary mutations but can be challenging, especially for patients with locally advanced disease and no easily accessible lesions. Identification of acquired resistance mutations in circulating tumor DNA (ctDNA), collected during or after platinum induction, can be helpful. Based on available data, patients with *BRCA2* reversion mutations should not be treated with PARPi maintenance; rather, 5-FU maintenance should be selected. For patients with initial benefit on PARPi maintenance, enrollment in trials with novel combinations aiming to bypass resistance, such as ATR or Wee1 inhibitors, should be encouraged.

If maintenance therapy after non-progression on platinum-based therapy remains the right place for PARPi in patients with *gBRCA1/2* patients, how can we improve on the results of POLO study? The activation of antitumor immunity with PARPi and the so far documented safety in combination with immune checkpoint inhibitors begs for evaluation after initial induction chemotherapy. A study with niraparib with nivolumab/ipilimumab is currently ongoing (NCT03404960) [103]. Furthermore, PARPi combinations with MEK or VEGFR inhibitors and or radiotherapy can potentially improve outcomes.

## 6. Conclusions

In conclusion, PARPi are potential therapeutic options in advanced PDAC in select patients with *gBRCA1/2* pathogenic mutations and platinum sensitivity. Combining PARPi with other classes of drugs poses the challenge of balancing the therapeutic benefit and potential overlapping toxicities. Besides careful selection of patients and drugs, testing the drugs sequentially rather than in combination could be another strategy to achieve an optimal risk vs. benefit ratio. Future efforts should entail a better understanding of the underlying mechanism of actions, resistance mechanisms, and biomarker development to achieve maximal therapeutic benefit at the cost of minimal side effects. A number of clinical trials are exploring this promising class of drugs in different patient populations.

## Figures and Tables

**Table 1:** Current PARP inhibitors under clinical development.

Agent	^#^ Potency for PARP Trapping	Mono Therapy Dose	Toxicities *		FDA Approvals ^∫^
			Any (%)	Grade >/3 (%)	
Olaparib	++	300 mg PO BID	Any 96% Fatigue 60% Nausea 45% Anemia 27% Abd pain 29% Diarrhea 29% Anorexia 25% Constipation 23%	Any 40% Fatigue 5% Anemia 11% Abd pain 2% Anorexia 3Vomiting 1% Arthralgia 1%	-2014: *gBRCA* mutation positive ovarian cancer in 4th line of therapy. ORR of 34%, PFS of 6.7 mo [56] -2017: Maintenance post-recurrence in ovarian, primary peritoneal or fallopian tube carcinoma with CR or PR after platinum-based chemotherapy irrespective of BRCA status. PFS: olaparib (19 mo) vs. placebo (5.5 mo), HR 0.3, *p* < 0.0001) [57,58] -2018: Front-line maintenance in *gBRCA* or *sBRCA* mutation positive ovarian or primary peritoneal or fallopian tube carcinoma with CR or PR after platinum-based chemotherapy. 3-yr OS: olaparib (84%) vs placebo (80%), HR 0.95 (95% CI 0.6–1.53) [59] -2018: *gBRCA* mutation positive, Her-2 negative metastatic breast cancer </2 lines of therapy. PFS: olaparib (7 mo) vs std Rx (4 mo), HR 0.58 (*p* < 0.001); OS: olaparib (19.3 mo) vs. standard therapy (19.6mo), HR 0.9 (*p* = 0.57) [60]
Rucaparib	++	600 mg PO BID	Any 100% Nausea 75% Fatigue 69% Dysgeusia 39% Anemia 37% Constipation, Vomiting 37% Transaminitis 34% Diarrhea 32% Abd pain 30% Thrombocytopenia 28%	Any 48% Anemia 18% Asthenia 7% Neutropenia 5% Thrombocytopenia 3% Transaminitis 10% Nausea, Vomiting 4% Abd pain, Diarrhea, Anorexia, Arthralgia 1%	-2016: *gBRCA* or *sBRCA* mutation positive ovarian cancer after >/2 lines of therapy. PFS: *BRCA* mutation positive (13 mo), LOH high (6 mo), LOH low (5.2mo) [61,62] -2018: Maintenance post-recurrence in ovarian or primary peritoneal or fallopian tube carcinoma with CR or PR after platinum-based chemotherapy PFS: rucaparib (17mo) vs placebo (5 mo), HR 0.23 (*p* < 0.0001) [63]
Veliparib	+	400 mg PO BID	Nausea 43% Vomiting 29% Anemia 24% Leukopenia 20% Thrombocytopenia 9%		
Niraparib	+++	300 mg PO QD	Nausea 74% Thrombocytopenia 61% Fatigue 59% Anemia 50% Constipation 40% Vomiting 34% Neutropenia 30% Headache 26% Anorexia 25% Insomnia 24% Abd pain 23%	Thrombocytopenia 34% Anemia 25% Neutropenia 20% Fatigue 8% Nausea 3% Hypertension 8% Vomiting 2% Abd pain, Dyspnea 1%	-2017: Maintenance post-recurrence in ovarian or primary peritoneal or fallopian tube carcinoma with CR or PR after platinum-based chemotherapy. PFS: niraparib (21mo) vs placebo (5.5 mo), HR 0.27 (95% CI 0.17–0.41) [64]
Talazoparib	++++	1 mg PO QD	Anemia 53% Fatigue 50% Nausea 49% Headache 32% Neutropenia 35% Thrombocytopenia 27% Vomiting 25%	Anemia 39% Neutropenia 18% Thrombocytopenia 11% Leukopenia 6% Lymphopenia 3% Fatigue, Headache, Vomiting, back pain, dyspnea 2%	-2018: *gBRCA* mutation, Her-2 negative metastatic breast cancer </3 lines of therapy. PFS: talazoparib (8.6mo) vs placebo (5.6 mo), HR 0.54; *p* < 0.001. Interim OS HR 0.76 (95% CI 0.55–1.06, *p* = 0.11) [65]
Pamiparib		60 mg PO BID	Nausea 50% Fatigue 33% Anemia 20% Vomiting 15% Neutropenia 13%	Anemia 13% Neutropenia 8% Fatigue 5%	No FDA approved indications yet

^#^ Relative PARP Trapping potency; +++ indicates the most potent, + indicates the least potent. ^∫^ As of 10th October 2019; * Olaparib based on Phase III POLO trial [66], Rucaparib based on Phase III ARIEL III [63], Niraparib based on Phase III NOVA trial [64], Talazoparib based on EMBRACA trial [65], Veliparib based on a Phase II trial [67], Pamiparib-Immature data from Phase I NCT 02361723 [55], Ongoing Phase III Pamiparib studies- NCT03519230, NCT03427814. Abbreviations: PARP = poly(ADP-ribose)polymerase; g = germline; s = somatic; m = mutant; CR = complete remission; PR = partial remission; Her-2 = Human epidermal growth factor receptor-2; Abd = abdominal; PFS = median progression free survival; OS= median overall survival.

**Table 2:** Trial results PARP inhibitors in pancreatic cancer

Clinical Study	Phase	Patient Population	Intervention	Outcome	ADEs
**PARPi as Monotherapy**					
NCT01078662 Kauffman et al. [56]	II	*gBRCA1/2* mutation positive advanced recurrent cancers, PDAC cohort with progression on gemcitabine (65% prior platinum-based regimen)	Single arm olaparib 400mg PO BID	*PEP:* ORR (PDAC cohort) 22% *SEP:* Stable disease at > 8 weeks 35%, DOR 134 days, PFS 4.6 mo, OS 9.8 mo	*Any grade*: Fatigue (74%), Nausea (48%), Vomiting, Anemia (40%) *Grade >/3*: Anemia (17%) Fatigue (13%)
NCT02042378 Shroff et al. [70]	II	*sBRCA1/2* or *gBRCA1/2* mutation positive advanced PDAC, 1–2 prior lines of therapy, prior platinum exposure in 79% pts, platinum refractory disease in 42% patients	Single arm rucaparib 600 mg BID	*PEP:* ORR 16% (3/19, 1CR and 2PR), *SEP:* DCR 32%, 44% with 1 prior line of therapy	*Any grade:* Nausea (63%) Anemia (47%), Abdominal pain, fatigue (37%) *Grade >/3:* Anemia (32%) Fatigue, Ascites (16%) Nausea, abdominal pain, increased AST, ALT (10%)
Lowery et al. [71]	II	*gBRCA1/2* or *PALB2* mutation positive advanced PDAC patients, prior 1–2 lines of therapies (88% prior platinum-based regimen, 64% of these pts had PD on platinum-based regimen)	Single arm veliparib 300mg BID PO (*n* = 3), 400 mg BID (*n* = 15)	*PEP:* ORR-No CR or PR, Stable disease 25% pts at 8 weeks *SEP:* PFS 1.7 mo, OS 3.1 mo	Fatigue (25%) Elevated bilirubin (19%) Thrombocytopenia, dehydration, increased alkaline phosphatase (13%)
NCT02677038, NCT02511223 Golan et al. [72]	II	Advanced PDAC, >/1 lines of therapy with *BRCAness* phenotype	Single arm olaparib PO BID	*PEP:* ORR Israel-5SD, 12 PD; U.S.-2PR, 6 SD, 3 PD *SEP:* PFS-14 wks (Israel) and 25 wks (U.S.)	Grade 1–2 anemia, fatigue, nausea
**PARPi as Maintenance Therapy**					
NCT02184195, Golan et al. [66]	III	*gBRCA1/2* mutation positive, mPDAC, non-progressive disease during first line platinum-based therapy for at least 16 weeks	3:2 randomization to olaparib versus placebo	*PEP:* PFS-7.4 mo vs 3.8 mo (HR 0.53, *p* = 0.004) *SEP:* OS (46% data maturity)-19 mo vs. 18 mo (*p* = 0.7), no difference in HrQOL scores	*Any grade:* Olaparib vs. placebo (96% vs. 93%), Fatigue (60% vs. 35%), nausea (45% vs. 23%), abdominal pain (29% vs. 25%), anemia (27% vs. 17%) *Grade >/3:* Olaparib vs placebo (40% vs. 23%) Anemia (11% vs. 3%) Fatigue (5% vs. 2%) Abdominal pain (2% each)
NCT 03140670, Binder et al. [73]	II	*gBRCA1/2*, *gPALB2*, *sBRCA1/2*, or *sPALB2* mutation positive advanced PDAC, non-progressive disease during first line platinum-based therapy for at least 16 weeks	Single arm rucaparib 600mg PO BID	*PEP:* PFS Prelim data (19/24 pts enrolled, 42 planned) -mPFS of 9 mo *SEP:* ORR 37% (1CR, 6 PRs), DCR-90% for at least 8 weeks	Most common (grade 1,2): Nausea (46%) Dysgeusia (33%) Fatigue (25%) No grade 3 ADEs
**PARP in combination with chemotherapy**					
NCT02890355, Chiorean et al. [74]	II	mPDAC, second line therapy with (1st line Rx with non-irinotecan-based therapy), 9% (11/123) pts with HRD (4 germline *BRCA1/2*, *ATM*; 7 somatic *BCRA2, PALB2, ATM, CDK12*), 20% (24/123) pts with DDR, non HRD mutations	1:1 randomization to either veliparib + FOLFIRI vs FOLFIRI alone	Planned interim futility analysis at 35% PFS events *PEP:* OS 5.1 vs. 5.9 mo (HR 1.3, *p* = 0.2) *SEP:* PFS 2 mo vs. 3 mo (HR 1.5, *p* = 0.05)	Most common Grade >/3 ADEs: Veliparib + mFOLFIRI vs FOLFIRI Neutropenia (33% vs. 20%) Fatigue (19% vs. 4%) Nausea (11% vs. 4%)
NCT01489865, Pishvaian et al. [75]	I/II	mPDAC pts, phase I (31 pts), phase II (33pts). Phase II pts preselected for germline or somatic *BRCA1/2, PALB2* mutations (27%) or FH of breast /ovarian syndrome (69%); both previously treated (18/33) and untreated (15/33)	Veliparib + mFOLFOX6	57/64 pts evaluable *PEP:* ORR-26% all pts, 58% in pts with +FH, +DDR, platinum naïve (12 pts) *SEP:* OS 8.5 mo, PFS 3.7 mo	*Grade >/3:* Myelosuppression (16%) Nausea, vomiting (6%)

Abbreviations: g = germline; s = somatic; mPDAC = metastatic pancreatic ductal adenocarcinoma; PEP = primary end point; SEP = secondary end point; ORR = overall response rate; CR = complete response; PR = partial response; PFS = progression free survival; OS = overall survival; DOR = duration of response; DCR = disease control rate; mo = months; ADE = adverse drug-related events; FOLFIRI = FOLinic Acid+ 5-Fluorouracil+ IRInotecan; FOLFOX = FOLinic acid+ 5-Fluorouracil + Oxaliplatin; FH = family history; DDR = defective DNA damage repair; abd = abdominal; PO = per os; PARP = poly(ADP-ribose) polymerase.
